# German Review

**Published:** 1891-05

**Authors:** Leo. Breisacher


### Selections from Foreign Journals.

### *GERMAN.*

By Leo. Breisacher, V. M. D.

### Antiseptic Properties of Coffee Infusion.

The investigations of Dr. Lüderitz the results of which were published in the Zeitschrift f. Hygiene, Bd. 7, Nr. 241, show that an infusion of coffee possesses decided antispeptic properties. Bacteria of every kind are destroyed by its influence. It has not been ascertained as yet through which chemical substance this action is exerted. Caffein seems to have very little influence, tannic has a somewhat stronger influence; the chief action however seems to depend upon the empyreumatic substances that develop through the roasting of the coffee. *Wochenschrift f. Thierheil. No. 47.*

---

### The Action of Various Drugs in Veterinary Practice.

Jahresberichten Bayrischer Thierarzte. 1889.

*Antefebrin:*—In distemper and the various febrile diseases, good results were gotten in the dog with 30 to 45 grains.

In horses or cattle doses of 20, grammes lower temperature from 1½ to 2 degrees.

*Apomorphin:* — In bronchitis is contra-indicated when at the beginning of the attack there exists an increased respiration.

In some cases doses of 0.1 grammes per day have caused excessive dyspnœa through its decided action upon the mucous secretion. This drug should be used with great care. Used subcutaneously in dogs it acts as a prompt emetic.

*Creolin:*—In traumatic lesions of the uterus, in acute and chronic metritis, in skin diseases of every kind and in diphtheritis of the chicken is highly recommended.

*Eseridin:*— In doses of 0.2 grammes pilocorpin-eserin has very little action. Doses of 0.1 grammes of eseridin, subcutaneously, exerts a decided action in 10 minutes, in horses. Its administration should be followed by a dose of aloes.

*Ichthyol* (Ammonium sulfo-ichthyolicum): — Has a very good action in articular rheumatism, applied externally. It has been used dissolved in glycerine 1 to 10 and in vaseline 1 to 5 parts.

*Sodium Chloride:* — Exerts a very good inflnence in cases of shoulder and hip lameness when injected subcutaneously—one part of concentrated nace to 2 or 3 of water.

*Liquor alumnii acetici:*—Is very highly recommended for the treatment of wounds and especially in the various affections of the uterus. It does not cause peristalsis of the uterus as does carbolic acid creolin and corrosive sublimate, and has no poisonous action. *Thierheilkunde 51, '90 to 3, '91.*

---

### Pyoktanin in Foot and Mouth Disease.

This substance which was discovered by Prof. Stilling--Strassburg, has given very good results in foot and mouth disease. Dr. Mehrdorf who from July 15 to November 15 1890 had occassion to use this substance in 1293 cases (swine cattle and goats) asserts that in his 20 years of practice he has not met with such an active agent for the combating of foot and mouth disease as pyoktanin.

The advantages of pyoktanin are:— 1st that the timely application will in the future prevent all losses; 2nd that through its use the condition of the animal is very little or not at all affected; 3rd that the cessation of the milk secretion lasts but a few days; 4th that the disease assumes a mild character; and 5th that draught animals are in short time after the attack capable of executing work again. The only objection to the remedy is that it stains the hands very intensely (according to the editor of Thierheilkunde soap dissolved in alcohol removes the color entirely) this property has however on the other hand the advantage that it shows exactly to what extent the remedy has been applied to the diseased parts. Dr. M. used as solution of 1 : 1000. See original article for details. *Wochenschrift f. Thierheilkunde No 4, 1891.*

---

### Diphtheritis in Poultry.

In Oct. a farmer sent to Veteriniary Thum two turkeys with the history that they were affected with loss of appetite or inability to eat. It was furthermore stated that the entire stock was likewise affected. The head and neck of the turkey examined showed numerous nodules or papules which lead immediately to the idea that he had to do with diphtheritis, and this surmise proved to be a correct one, for upon opening the bill of the fowl he discovered numerous diphtheritic patches. The animals were treated with external applications, one day with acid carbolic, 1 part to glycerine 100 parts. the following day with creolin 2 parts and glycerine and aq. distill., aa 50 parts. This treatment was continued for three weeks with the result that it cured every animal. *Wochenschrift f. Thierheilkunde No 55—'91.*

---

### The Treatment of Glanders With Mercury.

Dr. Tizer—Wogen (Deutsche Med. Zeitung No. 68, 1890.

In the Berl. Klin. Wochenschrift Dr. Holda published lately a case of glanders in man that had been successfully treated by means of the mercury treatment. T. in his paper gives three cases that were entirely cured by the mercury treatment. Case 1. a peasant complained of pain and a feeling of *heaviness* in the lower extremities, headache, dullness (Beklemmüng) and cough. Temp. was 38.9 Pulse 100. Auscultation of the lungs showed the existence of a fine crepitant rale while percussion gave only normal sounds. On the arms and legs there existed eruptions and excresences. The disease had existed for 8 days and the patient could not remember of having come in contact with horses. The patient was unable to give the faintest clue in regard to the disease. The bacteriological examination however did not leave T. in doubt in regard to the nature of the disease; it was glanders. As it was known to T. that the various drugs that are recommended in glanders are inefficacious, he decided to give mercury a trial. Inunctions of grey salve and a warm bath were given every two days, mouth, pharnyx etc. were treated with chlorate of potassium while internally quinine was given. The excresences or small tumors were opened and treated with iodoform and later they were treated with the Paquelin cautery to favor granulation. After the 68th inunction, in the course of three months, the patient recovered completely. One year the patient was kept under the observation of T.; a recurrence of the disease however did not set in. The two other cases also terminated successfully. Dr. Wechseler reported in a Russian journal a successful mercury cure. Whether all cases will respond to the treatment as did the cases numerated above, it is impossible to say, however the results gotten in these 5 cases indicate that the mercury treatment deserves at any rate a thorough trial. *Oesterreichische Monatschrift f. Thierheilkunde, No. 2 1891.*

---

### The Liebreich Remedy.

At a recent meeting of the Berl. Med. Gesellschaft, Liebreich made known the nature of the new panacea. We say panacea for he does not only claim that it will have a good action in tuberculosis but in every disease where there exists an altered function of the tissue cells. Liebreich noticed that the beneficial action of the Koch fluid in Lupus was preceded or accompanied by an intense cell formation. He thought it quite probable that this action was simply due to some stimulating properties of the lymph. To test the correctness of this idea he took from the shelf that ancient cantharadine that in human medicine had become enveloped in dust and oblivion, and its restoration caused no *little flurry*. The solution is made in the following manner; 0.3 hyrate of sodium and 0.2 of cantharidine are dissolved in a small quantity of water in a 1000 c. cm. flask on the water-bath. Under the influence of the heat of the water bath about 900 c. cm. of water are gradually added, then the flask removed, the fluid allowed to cool, and the flask filled to the 1000 mark. At first he used potassium but it was soon discovered that it exerted a too decided action on the circulation and the nervous system. *L. B.*

